# Follicular Output Rate and Follicle-to-Oocyte Index of Low Prognosis Patients According to POSEIDON Criteria: A Retrospective Cohort Study of 32,128 Treatment Cycles

**DOI:** 10.3389/fendo.2020.00181

**Published:** 2020-04-07

**Authors:** Lijuan Chen, Hui Wang, Hanying Zhou, Haiyan Bai, Tao Wang, Wenhao Shi, Juanzi Shi

**Affiliations:** The Assisted Reproduction Center, Northwest Women’s and Children’s Hospital, Xi’an Jiaotong University, Xi’an, China

**Keywords:** POSEIDON, low prognosis patient, ovarian sensitivity, follicular output rate, follicle-to-oocyte index

## Abstract

**Objective:**

To investigate ovarian sensitivity in subgroups of patients with a low prognosis, as defined by the POSEIDON criteria, undergoing *in vitro* fertilization treatment and measures to improve ovarian sensitivity in these patients.

**Design:**

We conducted a retrospective cohort analysis.

**Setting:**

The study was conducted at an IVF clinic in a public hospital.

**Patients:**

A total of 32,128 fresh IVF cycles from January 2014 to October 2018 at a single IVF clinic were included in the analysis. Patients with a low prognosis were categorized into four groups based on the POSEIDON criteria.

**Interventions:**

None.

**Main Outcome Measure:**

The primary outcome measures were the follicular output rate (FORT) and the follicle-to-oocyte index (FOI).

**Results:**

The FORTs in the order from the highest to the lowest were 1.18 in group 3, 0.98 in group 4, 0.76 in group 1, and 0.68 in group 2. The trend in the FOI values was consistent with that in the FORTs. Among patients with poor ovarian sensitivity, 58.41% of patients with FORTs ≥ 0.30 in the second cycle underwent an adjustment to the ovarian stimulation (OS) protocol and 41.59% underwent an adjustment to the gonadotropin (Gn) starting dose. Among patients with normal ovarian sensitivity, 43.56% of those with FORTs ≥ 0.80 in the second cycle underwent an adjustment to the OS protocol and 56.44% underwent an adjustment to the Gn starting dose.

**Conclusion:**

Ovarian sensitivity was the highest in group 3 (young women with poor ovarian reserve), followed by groups 4 (women at advanced age with poor ovarian reserve) and 1 (young women with good ovarian reserve), and it was the lowest in group 2 (women at advanced age with good ovarian reserve). For patients with poor ovarian sensitivity, it is preferred to recommend an adjustment to the OS protocol, while for those with normal ovarian sensitivity, adjusting the Gn starting dose is preferred.

## Introduction

The definition for patients with low ovarian response was different in the past, but these patients have received great attention. The Bologna criteria (BR1), proposed in 2011, defined the population of women with poor ovarian response (POR) as a single patient population, ignoring their heterogeneity and the impact of age-related oocyte quality. Although the Bologna criteria were the first clear criteria to identify poor responders, they could not indicate the most effective treatment or the underlying causes of low response. The 2016 POSEIDON Working Group proposed a new concept of “low prognosis” (BR2). The POSEIDON criteria is more systematically based than the Bologna criteria and can be used as a reference for clinical practice. The incidence of patients with low prognosis attending a fertility center might vary between clinics and countries, but studies indicate that 47% of patients who undergo Assisted Reproductive Technology (ART) fit into one of the POSEIDON categories (BR3).

Traditionally, the ovarian response is predicted based on age and ovarian reserve. Patients in the POSEIDON groups 1 and 2, despite having adequate ovarian markers, have a suboptimal or low number of oocytes retrieved. The follicular output rate (FORT) and follicle-to-oocyte index (FOI), which are considered qualitative markers of ovarian response, may most optimally reflect the dynamic nature of follicular growth in response to exogenous gonadotropin (Gn) (BR4). The FORT and FOI can also help to better understand patients who may benefit from pharmacological therapy to improve the oocyte yield (BR4). Lastly, the FORT and FOI are considered to be positively related to the outcomes of *in vitro* fertilization (IVF) (BR5). Until now, there have been few reports regarding the FORTs and FOI values among the four patient groups defined using the POSEIDON criteria.

The objective of this study was to characterize low prognosis patients in order to facilitate the treatment decision-making process. In this study, baseline characteristics of the patient groups defined using the POSEIDON criteria were analyzed, and the FORT and FOI values following one aspirated IVF/intracytoplasmic sperm injection (ICSI) cycle were proposed as the primary outcome measures for low prognosis patients undergoing IVF treatment.

## Materials and Methods

This was a retrospective study of 32,128 fresh IVF cycles from January 2014 to October 2018 in our center. Data were extracted from the electronic medical record system (Wuhan Huchuang Co., Ltd., Version 9.2.5.8). The study was approved by the Ethics Committee for the Clinical Application of Human Assisted Reproductive Technology of Northwest Women’s and Children’s Hospital (No. 2018002).

### Inclusion Criteria

Patients were categorized according to the POSEIDON criteria, as outlined below. Only those who received conventional ovarian stimulation (OS) in the first cycle were included.

Patients with low prognosis:

Group 1 (*n* = 1,787 cycles): age < 35 years; antral follicle count (AFC) ≥ 5; number of oocytes retrieved in the previous cycle ≤ 9.
Group 1a (*n* = 465 cycles): number of oocytes retrieved in the previous cycle < 4.
Group 1b (*n* = 1,322 cycles): number of oocytes retrieved in the previous cycle, 4–9.
Group 2 (*n* = 1,001 cycles): age ≥ 35 years; AFC ≥ 5; number of oocytes retrieved in the previous cycle ≤ 9.
Group 2a (*n* = 398 cycles): number of oocytes retrieved in the previous cycle < 4.
Group 2b (*n* = 603 cycles): number of oocytes retrieved in the previous cycle, 4–9.
Group 3 (*n* = 1,447 cycles): age < 35 years; AFC < 5.
Group 4 (*n* = 2,148 cycles): age ≥ 35 years; AFC < 5.

Patients with non-low prognosis:

Group 5 (*n* = 25,745 cycles): AFC ≥ 5; previous OS of >9 oocytes or no previous OS.

**FIGURE 1:**
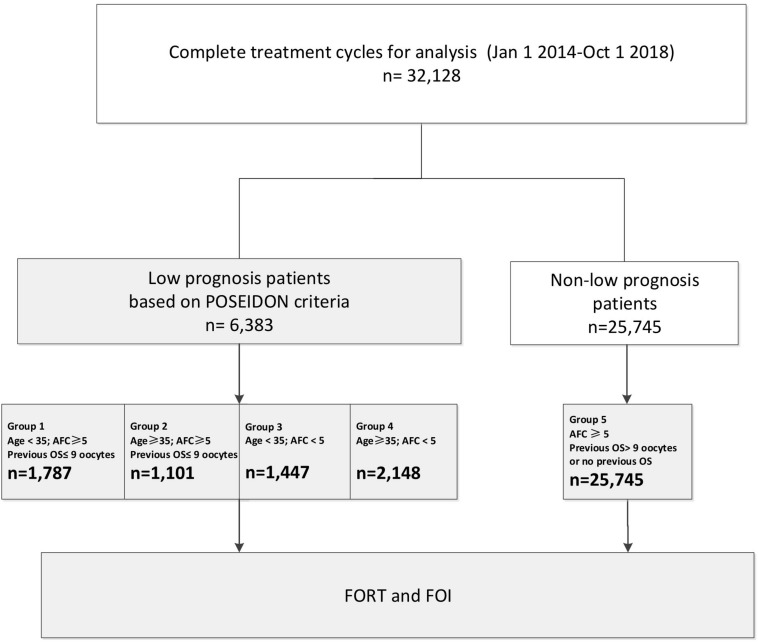
Flow chart and data processing.

The flow chart and data processing procedure are displayed in Figure 1. The demographics and baseline characteristics of patients are presented in Table 1.

**TABLE 1:** Demographics and baseline characteristics.

	**Low prognosis patients (LPPs)**				**Non-LPPs**	***P*-value**
**Group**	**1**	**2**	**3**	**4**	**5**	
*N*	1,787	1,001	1,447	2,148	25,745	
Age	29.8 (29.6, 29.9)	38.7 (38.5, 38.9)	30.2 (30.1, 30.4)	40.1 (39.9, 40.2)	29.9 (29.8, 29.9)	<0.001
BMI	22.0 (21.9, 22.2)	22.3 (22.1, 22.5)	22.1 (21.9, 22.3)	22.5 (22.4, 22.6)	22.2 (22.1, 22.2)	<0.001
Basal FSH (IU/ml)	7.5 (7.4, 7.7)	8.4 (8.2, 8.7)	8.8 (8.5, 9.0)	10.0 (9.8 10.2)	6.6 (6.6%)	<0.001
Type of infertility						<0.001
Primary	1,043 (58.4%)	228 (22.8%)	877 (60.6%)	429 (20.0%)	14,411 (56.0%)	
Secondary	744 (41.6%)	773 (77.2%)	570 (39.4%)	1,719 (80.0%)	11,334 (44.0%)	
Length of infertility, years	2.9 (2.8 3.0)	3.1 (2.9 3.3)	2.9 (2.8 3.1)	3.0 (2.9 3.2)	2.8 (2.8 2.9)	<0.001
AFC	9.2 (9.0 9.4)	7.3 (7.1 7.4)	3.0 (2.9 3.0)	2.8 (2.7 2.8)	12.2 (12.2 12.3)	<0.001
Smoking						0.041
Yes	11 (0.6%)	7 (0.7%)	15 (1.0%)	5 (0.2%)	149 (0.6%)	
No	1,776 (99.4%)	994 (99.3%)	1,432 (99.0%)	2,143 (99.8%)	25,596 (99.4%)	
OS protocol						<0.001
GnRH agonist	879 (49.2%)	347 (34.7%)	454 (31.4%)	547 (25.5%)	22,276 (86.6%)	
GnRH antagonist	730 (40.9%)	438 (43.8%)	560 (38.7%)	727 (33.9%)	3,073 (11.9%)	
Other	178 (10.0%)	216 (21.6%)	433 (29.9%)	874 (40.7%)	396 (1.5%)	
Gn type						<0.001
Recombinant-FSH	753 (42.5%)	225 (22.7%)	251 (17.8%)	143 (7.0%)	17,128 (66.6%)	
Urinary-FSH	1,019 (57.5%)	766 (77.3%)	1,162 (82.2%)	1,895 (93.0%)	8,572 (33.4%)	
Gn starting dose, IU	247.5 (243.0, 252.0)	291.2 (283.9, 298.6)	259.3 (254.4, 264.2)	286.2 (281.1, 291.4)	205.1 (203.9, 2.6.3)	<0.001

*BMI, body mass index; FSH, follicle-stimulating hormone; AFC, antral follicle count; OS, ovarian stimulation; GnRH, gonadotropin-releasing hormone; Gn, gonadotropin; FORT, follicular output rate; FOI, follicle-to-oocyte index. Median (95% CI)/*N* (%) calculated using EmpowerStats (www.empowerstats.com) and R. Kruskal-Wallis rank test for continuous variables; Chi-squared test for categorical variables; Fisher’s exact test for categorical variables with expected value < 10.*

### Exclusion Criteria

Patients receiving mild/natural IVF in the first cycle were excluded.

### Ovarian Stimulation and Oocyte Retrieval

The protocol for OS was determined individually according to the patients’ age, body mass index (BMI), basal follicle-stimulating hormone (FSH), and AFC. Of all 32,128 cycles, 29,104 comprised the first cycle and 3,024 comprised multiple cycles. All patients underwent a Gn-releasing hormone agonist or antagonist protocol followed by IVF or ICSI in the first cycle. Human chorionic gonadotropin (hCG; 4,000–10,000 IU) or recombinant hCG (r-hCG, MerckSerono S.p.A., 250 μg) was administered when 2–3 follicles increased to ≥17 mm. Oocytes were retrieved using transvaginal ultrasound-guided aspiration 36 h after hCG administration.

For patients with low ovarian sensitivity in the first cycle, most of them switched from agonist to antagonist, or antagonist to mild stimulation protocol. For patients with a small Gn starting dose in the previous cycle, we will increase the Gn starting dose, such as from 150 to 250 IU or 300 IU.

### FORT and FOI Definitions

The first primary outcome was the FORT, defined as the ratio between the number of pre-ovulatory follicles obtained in response to FSH administration and pre-existing pool of small antral follicles (BR5). Another primary outcome was the FOI, assessed as the ratio between the number of oocytes retrieved at oocyte pick-up and number of antral follicles on initiation of stimulation (BR4). In this study, the FORTs were divided into three groups: FORTs < 0.30 indicated low ovarian sensitivity; FORTs 0.30–0.80, normal ovarian sensitivity; FORTs > 0.80, high ovarian sensitivity. Similarly, the FOI values were divided into two groups: FOI values ≤ 0.50 indicated low ovarian sensitivity and those > 0.50 indicated normal ovarian sensitivity (BR4). The FORT and FOI values were calculated as follows:

FORT=Pre-ovulatory⁢follicle⁢count⁢on⁢dhCG÷AFC

FOI=The⁢number⁢of⁢oocytes⁢retrieved⁢at⁢oocyte⁢pick-up÷AFC

### Statistical Analysis

The data processing and statistical analysis were performed using EmpowerStats software^1^ and the statistical software package R. Kruskal-Wallis rank test was performed for continuous variables; chi-squared test was performed for categorical variables; and Fisher’s exact test was performed for categorical variables with expected value < 10. To assess the odds ratios (ORs) of the FORT and FOI in different patient groups, a multivariate regression model was established, using potential confounding factors as variables and was adjusted for age, BMI, basal FSH, AFC, OS protocol, Gn type and Gn starting dose. The Pearson correlation test was performed to determine the correlation between FOI values and FORTs. A value of *P* < 0.05 was considered statistically significant.

## Results

**FIGURE 2:**
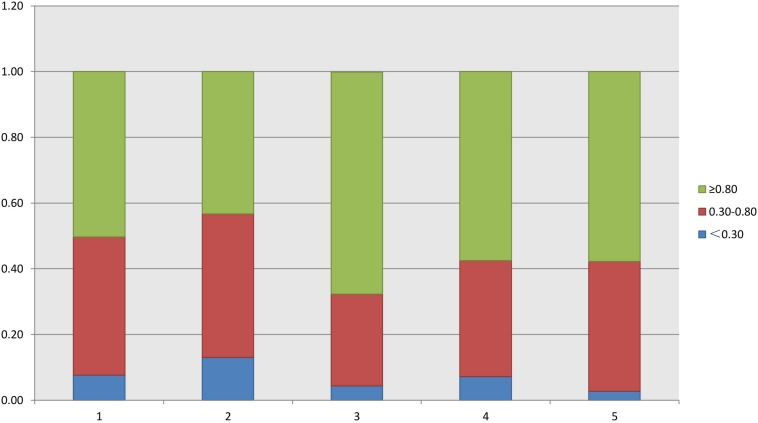
Distribution of FORT in each group.

**FIGURE 3:**
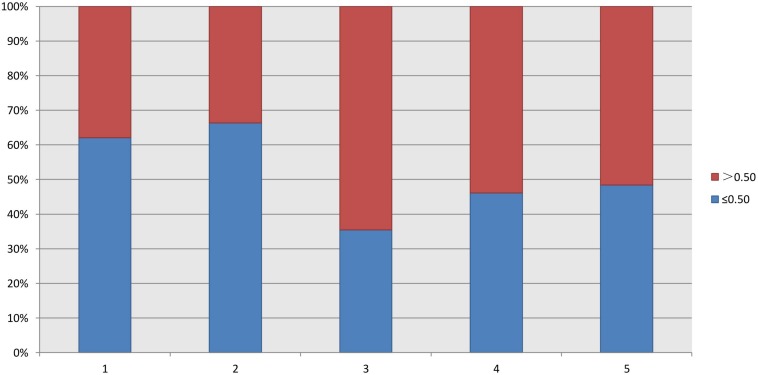
Distribution of FOI in each group.

As shown in Table 2, the FORTs in the order from the highest to the lowest were 1.18 in group 3, 0.98 in group 4, 0.76 in group 1 and 0.68 in group 2. The trend in the FOI values was consistent with that in the FORTs. The distribution of FORTs and FOI values among the five study groups is shown in Figures 2, 3. The highest proportion of patients with FORTs ≥ 0.30 was in group 5, followed by in groups 3, 4, 1, and 2. The patient proportion trend in FOI values > 0.50 was similar to that in FORTs. FORTs and FOI values of the POSEIDON subgroups 1a, 1b, 2a, and 2b were shown in Table 3. FORTs and FOI values in the different POSEIDON groups according to the use of the OS protocol and the type of Gn and hCG were shown in Tables 4–6.

**TABLE 2:** FORT and FOI of each group.

	**Low prognosis patients (LPPs)**				**Non-LPPs**	***P*-value**
**Group**	**1**	**2**	**3**	**4**	**5**	
*N*	1,787	1,001	1,447	2,148	25,745	
FORT	0.76 (0.74, 0.78)	0.68 (0.65, 0.70)	1.18 (1.14, 1.21)	0.98 (0.96, 1.01)	0.84 (0.84, 0.85)	<0.001
FOI	0.69 (0.67, 0.71)	0.62 (0.59, 0.64)	1.07 (1.03, 1.11)	0.89 (0.86, 0.91)	0.83 (0.83, 0.84)	<0.001

*FORT, follicular output rate; FOI, follicle-to-oocyte index. Arithmetic mean (95% CI)/N (%) calculated using EmpowerStats (www.empowerstats.com) and R. Kruskal-Wallis rank test for continuous variables.*

**TABLE 3:** FORT, FOI of POSEIDON subgroups 1a, 1b, 2a, and 2b.

**Subgroup**	**1a**	**1b**	**2a**	**2b**	***P*-value**
*N*	465	1,322	398	603	
FORT	0.66 (0.62, 0.70)	0.79 (0.77, 0.82)	0.56 (0.53, 0.60)	0.77 (0.74, 0.80)	<0.001
FOI	0.58 (0.54, 0.62)	0.74 (0.72, 0.76)	0.49 (0.46, 0.52)	0.72 (0.68, 0.75)	<0.001

*FORT, follicular output rate; FOI, follicle-to-oocyte index. Arithmetic mean (95% CI) / N (%) calculated using EmpowerStats (www.empowerstats.com) and R. Kruskal-Wallis rank test for continuous variables.*

**TABLE 4:** FORT and FOI in different POSEIDON groups according to the use of agonist vs. antagonist protocol.

		**FORT**	***P*-value**	**FOI**	***P*-value**
Group 1	Agonist	0.79 (0.76, 0.82)	0.376	0.72 (0.69, 0.75)	1.000
	Antagonist	0.76 (0.73, 0.79)		0.71 (0.68, 0.75)	
Group2	Agonist	0.76 (0.72, 0.81)	0.760	0.68 (0.64, 0.72)	0.463
	Antagonist	0.73 (0.69, 0.77)		0.69 (0.65, 0.73)	
Group3	Agonist	1.37 (1.29, 1.44)	0.034	1.23 (1.16, 1.31)	0.889
	Antagonist	1.28 (1.22, 1.35)		1.23 (1.17, 1.30)	
Group4	Agonist	1.20 (1.14, 1.27)	0.132	1.05 (0.99, 1.11)	0.966
	Antagonist	1.12 (1.07, 1.17)		1.02 (0.97, 1.07)	
Group5	Agonist	0.85 (0.84, 0.85)	<0.001	0.84 (0.83, 0.84)	<0.001
	Antagonist	0.85 (0.84, 0.87)		0.83 (0.82, 0.85)	
Total			0.04		0.008

*FORT, follicular output rate; FOI, follicle-to-oocyte index. Arithmetic mean (95% CI)/N (%) calculated using EmpowerStats (www.empowerstats.com) and R. Kruskal-Wallis rank test for continuous variables.*

**TABLE 5:** FORT and FOI in different POSEIDON groups according to the type of Gn.

		**FORT**	***P*-value**	**FOI**	***P*-value**
Group 1	R-FSH	0.81 (0.78, 0.84)	<0.001	0.75 (0.72, 0.78)	0.001
	U-FSH	0.73 (0.70, 0.75)		0.66 (0.64, 0.69)	
Group 2	R-FSH	0.76 (0.71, 0.82)	0.004	0.70 (0.65, 0.76)	0.004
	U-FSH	0.67 (0.64, 0.69)		0.60 (0.57, 0.63)	
Group 3	R-FSH	1.49 (1.39, 1.59)	<0.001	1.41 (1.31, 1.52)	< 0.001
	U-FSH	1.13 (1.09, 1.17)		1.02 (0.98, 1.06)	
Group 4	R-FSH	1.30 (1.17, 1.43)	<0.001	1.15 (1.03, 1.28)	<0.001
	U-FSH	1.00 (0.97, 1.02)		0.89 (0.86, 0.92)	
Group 5	R-FSH	0.85 (0.84, 0.85)	0.010	0.85 (0.84, 0.85)	0.002
	U-FSH	0.83 (0.82, 0.84)		0.80 (0.79, 0.81)	
Total			0.040		<0.001

*FORT, follicular output rate; FOI, follicle-to-oocyte index; R-FSH, recombinant follicle-stimulating hormone; U-FSH, urinary follicle-stimulating hormone. Arithmetic mean (95% CI) / N (%) calculated using EmpowerStats (www.empowerstats.com) and R. Kruskal-Wallis rank test for continuous variables.*

**TABLE 6:** FORT and FOI in different POSEIDON groups according to the type of hCG.

		**FORT**	***P*-value**	**FOI**	***P*-value**
Group 1	R-hCG	0.98 (0.94, 1.02)	<0.001	0.75 (0.72, 0.78)	<0.001
	U-hCG	0.69 (0.66, 0.71)		0.66 (0.64, 0.69)	
Group 2	R-hCG	0.95 (0.88, 1.01)	<0.001	0.70 (0.65, 0.76)	<0.001
	U-hCG	0.65 (0.62, 0.67)		0.60 (0.57, 0.63)	
Group 3	R-hCG	2.14 (2.02, 2.26)	<0.001	1.41 (1.31, 1.52)	<0.001
	U-hCG	1.11 (1.07, 1.14)		1.02 (0.98, 1.06)	
Group 4	R-hCG	1.78 (1.63, 1.94)	<0.001	1.15 (1.03, 1.28)	<0.001
	U-hCG	0.96 (0.93, 0.99)		0.89 (0.86, 0.92)	
Group 5	R-hCG	0.94 (0.93, 0.95)	<0.001	0.85 (0.84, 0.85)	<0.001
	U-hCG	0.74 (0.73, 0.75)		0.80 (0.79, 0.81)	
Total			<0.001		<0.001

*FORT, follicular output rate; FOI, follicle-to-oocyte index; R-hCG, recombinant human chorionic gonadotropin; U-hCG, urinary human chorionic gonadotropin. Arithmetic mean (95% CI) / N (%) calculated using EmpowerStats (www.empowerstats.com) and R. Kruskal-Wallis rank test for continuous variables.*

**FIGURE 4:**
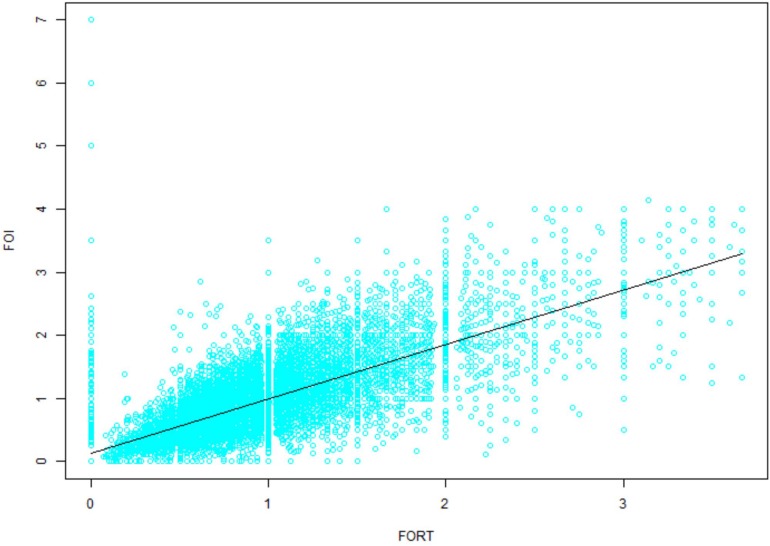
The correlation between FOI values and FORTs.

Multivariate regression analysis was performed using variables that could act as confounding factors, such as age, BMI, basal FSH, AFC, OS protocol, Gn type and Gn starting dose. The adjusted ORs of the FORTs and FOI values with 95% confidence intervals (CIs) are shown in Tables 7, 8. Consistent with the trend of non-adjusted results, the FORTs and FOI values in groups 3 and 4 were significantly higher than those in group 5. The FORTs in group 2 were the lowest (OR −0.13, 95% CI −0.18 to −0.08, *p* < 0.001). The correlation between FOI values and FORTs is shown in Figure 4. Data showed that FORTs and FOI values were positively correlated. The Pearson correlation coefficient was 0.779 (95% CI 0.775–0.784, *p* < 0.001).

**TABLE 7:** Multiple regression analysis for FORT.

	**Non-adjusted OR (95% CI), *P* value**	**Adjusted OR (95% CI), *P* value**
**Group**		
5	0	0
1	−0.07 (−0.09, −0.04), *p* < 0.001	−0.07 (−0.10, −0.04), *p* < 0.001
2	−0.14 (−0.17, −0.11), *p* < 0.001	−0.13 (−0.18, −0.08), *p* < 0.001
3	0.47 (0.44, 0.49), *p* < 0.001	0.35 (0.31, 0.38), *p* < 0.001
4	0.25 (0.23, 0.27), *p* < 0.001	0.24 (0.20, 0.28), *p* < 0.001

*OR, odds ratio. OR was adjusted for the age, BMI, basal FSH, AFC, OS protocol, Gn type and Gn starting dose.*

**TABLE 8:** Multiple regression analysis for FOI.

	**Non-adjusted OR (95% CI), *P* value**	**Adjusted OR (95% CI), *P* value**
**Group**		
5	0	0
1	−0.13 (−0.15, −0.10), *p* < 0.001	−0.14 (−0.18, −0.10), *p* < 0.001
2	−0.20 (−0.24, −0.17), *p* < 0.001	−0.14 (−0.19, −0.08), *p* < 0.001
3	0.33 (0.30, 0.36), *p* < 0.001	0.23 (0.18, 0.27), *p* < 0.001
4	0.10 (0.07, 0.12), *p* < 0.001	0.11 (0.07, 0.16), *p* < 0.001

*OR, odds ratio. OR was adjusted for the age, BMI, basal FSH, AFC, OS protocol, Gn type and Gn starting dose.*

**FIGURE 5:**
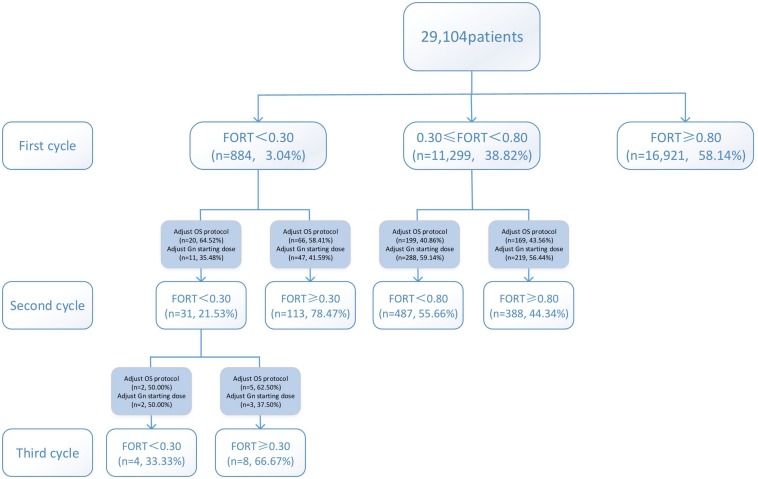
Variations in multi-cycle FORTs in the whole group of 29,104 patients.

**FIGURE 6:**
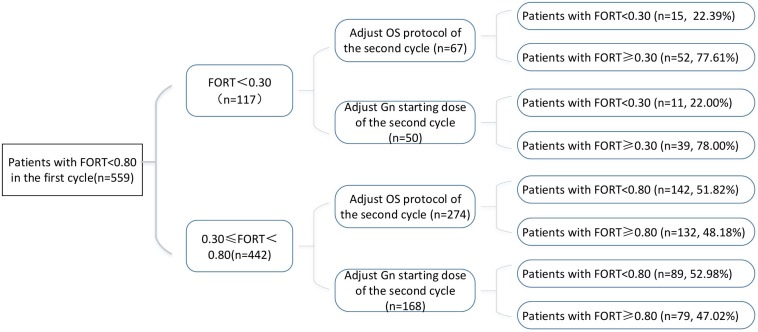
Variations in multi-cycle FORTs in patients with low prognosis.

**FIGURE 7:**
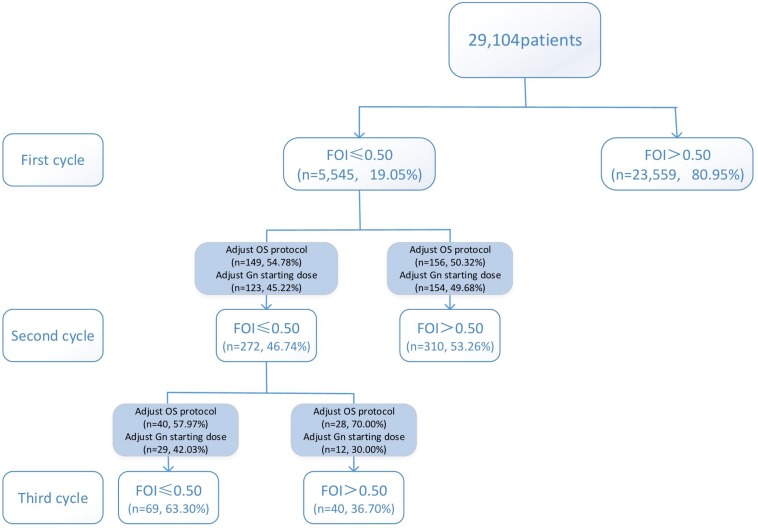
Variations in multi-cycle FOI values in the whole group of 29,104 patients.

Variations in multi-cycle FORTs and FOI values in the whole group of 29,104 patients are shown in Figures 5, 7. In the first cycle, 884 patients had FORTs < 0.30, the incidence of which was 3.04%. For patients with FORTs < 0.30, 58.41% of those with FORTs ≥ 0.30 in the second cycle underwent an adjustment to the OS protocol and 41.59% underwent an adjustment to the Gn starting dose. For patients with 0.30 ≤ FORT < 0.80, 43.56% of patients with FORTs ≥ 0.80 in the second cycle underwent an adjustment to the OS protocol and 56.44% underwent an adjustment to the Gn starting dose. After adjusting the OS protocol or Gn starting dose in patients with FORTs < 0.30 (*n* = 144), 31 patients (31/144, 21.53%) still had FORTs < 0.30 in the second cycle. Four patients had FORTs < 0.30 in all three cycles. The variation in multi-cycle FORTs in patients with low prognosis is shown in Figure 6. For patients with FORTs < 0.30 in the first cycle (*n* = 117), the probability of having FORTs ≥ 0.30 in the second cycle was 77.61% (52/67) after adjusting the OS protocol, while that of having FORTs ≥ 0.30 in the second cycle was 78.00% (39/50) after adjusting the Gn starting dose.

## Discussion

The central finding of the present study in a POSEIDON criteria-defined population was that the FORTs and FOI values were the highest in group 3, followed by in groups 4 and 1, and were the lowest in group 2. According to our results, although patients in group 2 had better ovarian reserve and more oocytes, the FORTs, and FOI values were lower in these patients than in those in group 3 with poorer ovarian reserve and fewer oocytes. The FORTs and FOI values in young women with diminished ovarian reserve (group 3) were slightly higher than those in young women with normal ovarian reserve (group 1).

The POSEIDON criteria aim to identify and stratify low prognosis patients into four distinct groups based on age, AFC and ovarian response in the previous cycle (BR6). POSEIDON groups 1 and 2 include patients with an unexpectedly suboptimal or low oocyte number despite adequate ovarian reserve. As such, these patients are expected to have lower ovarian sensitivity than those in groups 3 and 4. In the latter, the reason for the reduced number of oocytes retrieved relates to the low ovarian reserve.

Our results showed that in POSEIDON classification, older women (groups 2 and 4) had lower ovarian sensitivity than young women with diminished ovarian reserve (group 3). The primary reasons were the polymorphisms, low Gn dosage, suboptimal trigger, problems during oocyte pick-up. Older women also had impaired mitochondrial function, increased granulosa cell apoptosis, and increased oxidative stress. There is a hypothesis that the minimal responsiveness of antral follicles to exogenous FSH reveals oocyte dysfunction to a certain extent (BR7). The low FOI values in group 2 are expected because these patients may be hyporesponsive to the Gn dose or regimen due to factors such as polymorphisms.

In terms of the management of patients in group 2, greater attention should be paid to developing strategies to improve the oocyte quality rather than the oocyte quantity (BR8). For group 2 multi-cycle patients with poor ovarian sensitivity in the previous cycle, adjustment to the OS protocol is recommended first, followed by adjusting the Gn starting dose. This is consistent with findings of previous studies (BR9, BR10). Older women, in whom luteinizing hormone (LH) activity is insufficient, may notably benefit from additional LH (BR2, BR3). Supplements, such as growth hormone, dehydroepiandrosterone (DHEA), coenzyme Q10 and multi-nutrients, have been administered in an attempt to improve oocyte quality (BR11–BR14), although there is insufficient evidence to support their use in these patients.

Patients in group 3 had poor ovarian reserve and were expected poor responders. Interestingly, the FORTs and FOI values in group 3 were significantly higher compared to the other groups, although the Gn starting dose was not significantly increased in group 3 (Table 1). This suggests that the response of antral follicles to Gn reached the limit of its ability; therefore, there would be no additional benefit in regard to ovarian sensitivity to further increasing the daily Gn dose (BR15). Not all patients are insensitive to Gn, and the Gn dose can be increased appropriately, with a maximum daily dose of 300 IU (BR16). For group 3 multi-cycle patients with poor ovarian sensitivity in the previous cycle, adjustment to the OS protocol was equally effective as adjusting the Gn starting dose.

This study showed that the incidence of FORTs < 0.30 in patients undergoing ART was 3.04%. Among these patients, 58.41% of patients with FORTs ≥ 0.30 in the second cycle underwent an adjustment to the OS protocol and 41.59% underwent an adjustment to the Gn starting dose. Normal ovarian sensitivity was considered to be 0.30 ≤ FORT < 0.80, the incidence of which was 38.82%. Among these patients, 43.56% of patients with FORTs ≥ 0.80 in the second cycle underwent an adjustment to the OS protocol and 56.44% underwent an adjustment to the Gn starting dose. Therefore, for patients with poor ovarian sensitivity, it is preferred to recommend an adjustment to the OS protocol, while for those with normal ovarian sensitivity, adjusting the Gn starting dose is preferred.

The presence of genetic mutations or single nucleotide polymorphisms of Gn and their receptors can influence ovarian sensitivity to Gn stimulation (BR17, BR18). Apart from genotypic traits, there is also evidence to suggest that environmental contaminants and oxidative stress are involved in the hyporesponse pathogenesis (BR19–BR21). In this study, there were four multi-cycle patients in whom the FORT was still <0.30 after adjusting the OS protocol or Gn starting dose. This phenomenon was thought to be related to receptor polymorphisms or genetic mutations. It is recommended to conduct corresponding genetic screening. Wang et al. found a series of genetic mutations related to oocyte abnormalities, such as TUBB8, PANX1 and WEE2 (BR22–BR24). As research progresses, more genes related to oocyte abnormalities are anticipated to be discovered in succession.

The results of this study may open new possibilities in the treatment of low prognosis patients. The FORTs and FOI values were extremely high in group 3 than in the other groups, suggesting that we should attempt more OS protocol adjustments instead of blindly increasing the Gn dose. Limitations of this study are related to its retrospective nature, analysis from a single center, and heterogeneity in the OS protocols adopted.

## Conclusion

In conclusion, the FORTs and FOI values were the highest in group 3 (young women with poor ovarian reserve), followed by in groups 4 (older women with poor ovarian reserve) and 1 (young women with good ovarian reserve), and were the lowest in group 2 (older women with good ovarian reserve). Improving follicular responsiveness to Gn may be key to ameliorating the prognosis of patients with a low prognosis. The criteria for controlled ovarian hyperstimulation cancelation should be based on the output of the follicular response to exogenous Gn, rather than on the absolute count of follicles recruited by treatment. For patients with poor ovarian sensitivity, it is preferred to recommend an adjustment to the OS protocol, while for those with normal ovarian sensitivity, adjusting the Gn starting dose is preferred. For patients with multiple cycles of poor ovarian sensitivity, relevant genetic screening is recommended.

## Data Availability Statement

All datasets generated for this study are included in the article/supplementary material.

## Ethics Statement

The studies involving human participants were reviewed and approved by the Ethics Committee for the Clinical Application of Human Assisted Reproductive Technology of Northwest Women’s and Children’s Hospital. The patients/participants provided their written informed consent to participate in this study.

## Author Contributions

LC and JS conceived and designed the study. LC and WS drafted and revised the manuscript. HW, WS, and HZ analyzed and interpreted the data. HB and TW collected and cleared the data. All authors listed have contributed to the work and approved the final version.

## Conflict of Interest

The authors declare that the research was conducted in the absence of any commercial or financial relationships that could be construed as a potential conflict of interest.

## Footnotes

1 www.empowerstats.com
